# The Post-amyloid Era in Alzheimer's Disease: Trust Your Gut Feeling

**DOI:** 10.3389/fnagi.2019.00143

**Published:** 2019-06-26

**Authors:** Carolina Osorio, Tulasi Kanukuntla, Eddie Diaz, Nyla Jafri, Michael Cummings, Adonis Sfera

**Affiliations:** ^1^Psychiatry, Loma Linda University, Loma Linda, CA, United States; ^2^Department of Psychiatry, Patton State Hospital, San Bernardino, CA, United States

**Keywords:** microbiome, amyloid hypothesis, infection, senescence, inflammation

## Abstract

The amyloid hypothesis, the assumption that beta-amyloid toxicity is the primary cause of neuronal and synaptic loss, has been the mainstream research concept in Alzheimer's disease for the past two decades. Currently, this model is quietly being replaced by a more holistic, “systemic disease” paradigm which, like the aging process, affects multiple body tissues and organs, including the gut microbiota. It is well-established that inflammation is a hallmark of cellular senescence; however, the infection-senescence link has been less explored. Microbiota-induced senescence is a gradually emerging concept promoted by the discovery of pathogens and their products in Alzheimer's disease brains associated with senescent neurons, glia, and endothelial cells. Infectious agents have previously been associated with Alzheimer's disease, but the cause vs. effect issue could not be resolved. A recent study may have settled this debate as it shows that gingipain, a *Porphyromonas gingivalis* toxin, can be detected not only in Alzheimer's disease but also in the brains of older individuals deceased prior to developing the illness. In this review, we take the position that gut and other microbes from the body periphery reach the brain by triggering intestinal and blood-brain barrier senescence and disruption. We also surmise that novel Alzheimer's disease findings, including neuronal somatic mosaicism, iron dyshomeostasis, aggressive glial phenotypes, and loss of aerobic glycolysis, can be explained by the infection-senescence model. In addition, we discuss potential cellular senescence targets and therapeutic strategies, including iron chelators, inflammasome inhibitors, senolytic antibiotics, mitophagy inducers, and epigenetic metabolic reprograming.

**Graphical Abstract:**
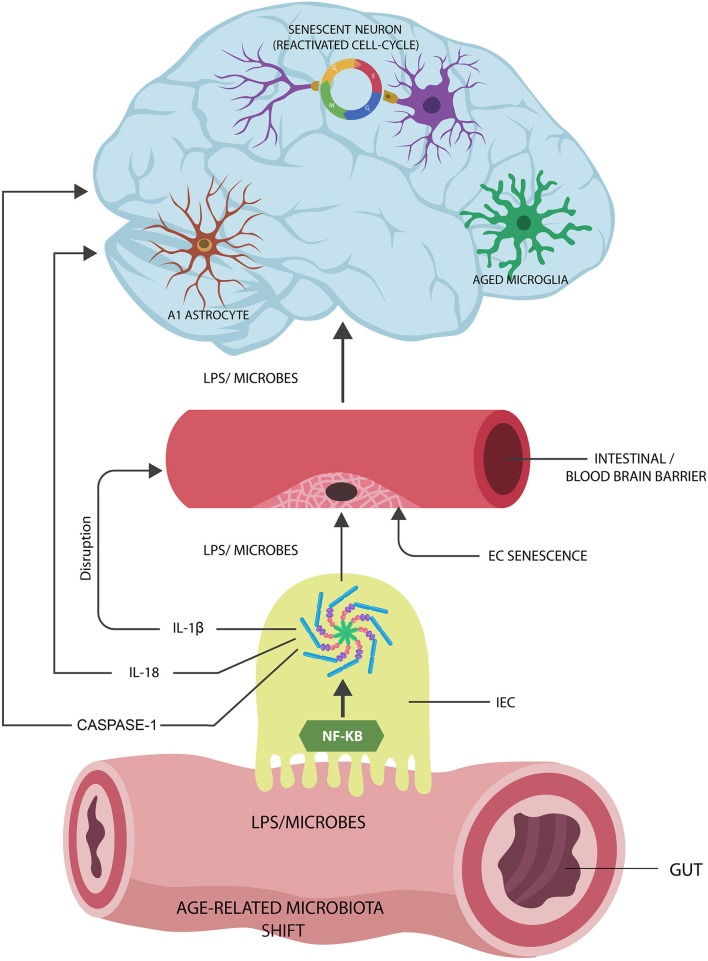
Proposed Alzheimer's disease (AD) pathogenesis: (1) Age-related gut microbiota shift leads to the upregulation of inflammagenic, lipopolysaccharide (LPS)-shedding microbial species. (2) These microorganisms activate nuclear factor kappa-light-chain-enhancer of activated B cells (NF-kB) and NOD-like receptor family pyrin domain-containing 3 (NLRP3) inflammasomes in intestinal epithelial cells (IECs), generating interleukin-1β (IL-1β), IL-18, and caspase-1. (3) IL-1β increases the permeability of intestinal and blood-brain barrier, allowing pathogen translocation into the body tissues and organs, including the brain. (4) Microorganisms and LPS induce cellular senescence in neurons, microglia, and astrocyte AD pathology.

## Introduction

Alzheimer's disease (AD) is the most common cause of dementia, affecting an estimated 5.5 million people in the US alone (Mayeux and Stern, 2012). Advanced age is a major AD risk factor; therefore, understanding cellular senescence and its impact on endothelial cells (ECs), neurons, glia, and immune cells is an essential prerequisite for elucidating the pathogenesis of this condition (Wiseman et al., 2018).

Brain accumulation of extracellular β-amyloid and intracellular hyperphosphorylated tau are the pathological hallmarks of AD. Both neurons and astrocytes synthesize β-amyloid from amyloid precursor protein (APP), while phagocytic microglia prevent its accumulation by removing it via the triggering receptor expressed on myeloid cells-2 (TREM-2) (discussed in the section “Beta Amyloid: Friend or Foe”).

Aging has been associated with pathological changes in microglia and astrocytes, including loss of neurotrophic properties and gain of toxic functions. These age-related glial alterations may contribute to AD pathology, marked by neuronal loss and memory impairment (discussed at length in the section “Senescent Astrocytes and Microglia”).

The amyloid hypothesis postulates that accumulation and deposition of β-amyloid are the primary causes of AD, which promotes tau aggregation into neurofibrillary tangles (NFTs), ultimately triggering neuronal death (Hardy and Allsop, 1991; Wildsmith et al., 2013). Although never universally accepted, the amyloid hypothesis drove AD research for at least two decades. Lately, however, many researchers and clinicians have questioned this model as amyloid removal failed to improve memory in numerous clinical trials (Fülöp et al., 2018a). With the same token, neuroimaging studies detected significant β-amyloid deposits in 20–30% of healthy older individuals, while in many AD patients, this marker was not observed (Edison et al., 2007; Li et al., 2008; Rodrigue et al., 2013; Higashi et al., 2018). Moreover, β-amyloid was recently characterized as an antimicrobial peptide (AMP), and its accumulation in AD brains may be a reflection of increased microbial burden (Alonso et al., 2018; Fülöp et al., 2018a). AMPs are defensive biomolecules secreted by the innate immune system, including microglia and astrocytes, in response to a variety of microorganisms and malignant cells (Alonso et al., 2018). The β-amyloid-AMP connection is further supported by the observation that central nervous system (CNS) infections were diagnosed in some clinical trials, following the administration of anti-amyloid vaccines (Orgogozo et al., 2003; Brothers et al., 2018; Zhan et al., 2018).

Recent studies have reported co-localization of microorganisms with senescent neurons and glial cells in the brains of both AD patients and healthy older individuals, reviving the infectious hypothesis entertained by Alois Alzheimer himself (De Chiara et al., 2012; Bester et al., 2015; Itzhaki et al., 2016; Alonso et al., 2018; Fulop et al., 2018b; Kritsilis et al., 2018).

CNS infectious agents have been detected previously in AD patients; however, it was difficult to assess if they represented the cause or effect of this condition (Hill et al., 2014). A recent study may have settled this issue as it detected gingipain, a *Porphyromonas gingivalis* antigen, linked to AD, in the brains of healthy older persons, suggesting that they would have developed the disease if they lived longer (Dominy et al., 2019). As *P. gingivalis* is a major cause of gum disease and a modifiable AD risk factor, treatment of periodontal infection must be considered a clinical priority.

A new study identified the disruption of the blood-brain barrier (BBB) as an early aging and AD marker, suggesting a portal for microbial brain entry (Montagne et al., 2015; Nation et al., 2019). Moreover, in stroke, microorganisms were shown to directly induce EC senescence and BBB disruption, carving an entry route into the CNS (Muller et al., 2009; Saito et al., 2010; Yamazaki et al., 2016; Aguilera et al., 2018).

Aside from how microbes enter the brain, identifying their source is essential for the development of new treatments. Recent studies have demonstrated elevated levels of microbes and lipopolysaccharide (LPS) in the CNS of both healthy elderly and AD patients, suggesting the gut as their point of origin (Zhao et al., 2017; Kowalski and Mulak, 2019). Interestingly, the gut microbial shift in older individuals is characterized by the increased preponderance of Gram-negative LPS-generating microbes, pointing to the gastrointestinal (GI) tract as the potential source of brain pathogens (Kobayashi et al., 2013; Sato S. et al., 2014; Greiner and Bäckhed, 2016; Odamaki et al., 2016; Yamazaki et al., 2016; Lebrun et al., 2017; Ke et al., 2018). Furthermore, loss of immune tolerance to commensal flora in older individuals and intestinal barrier disruption suggest the gut as the likely reservoir of brain LPS and microbes (Nagpal et al., 2018) (discussed in “The Senescent Intestinal Barrier”).

At the molecular level, cellular senescence has been associated with the activation of nuclear factor kappa-light-chain-enhancer of activated B cells (NF-kB) and NOD-like receptor family pyrin domain-containing 3 (NLRP3) inflammasomes (Yamazaki et al., 2016; Zhang W. et al., 2017; Burton and Stolzing, 2018). NLRP3 end products IL-18 and caspase-1 are associated with AD pathogenesis, while interleukin-1β (IL-1β) is an established disruptor of the BBB, linking it to microbial brain access (discussed in detail in “Senescence and Inflammasomes” section). In addition, activated NLRP3 inhibits autophagy and mitophagy (selective mitochondrial autophagy), contributing to inflammaging as the accumulation of senescent cells and damaged organelles triggers inflammation (Argaw et al., 2006; Bossù et al., 2010; Sutinen et al., 2012; Wang et al., 2014; Kim et al., 2016). Conversely, mitophagy enhancers deactivate NLRP3, limiting both cellular senescence and AD pathology (Gurung et al., 2014).

Microbiota-induced brain cells' senescence may explain other novel AD findings, including age-related neuronal genomic variation, aneuploidy, or somatic mosaicism (Argaw et al., 2006; Bossù et al., 2010). Senescent neurons reentering the cell cycle, a hallmark of AD, may account for this phenomenon, especially when apoptosis is inactivated (Paquola et al., 2016; McConnell et al., 2017; Sharma et al., 2017; Bai, 2018; Verheijen et al., 2018) (discussed in “Senescent Neurons and the Cell Cycle” section).

Senescent glial cells, probably including A1 astrocytes, have been associated with AD as they display neurotoxic functions, engaging in the elimination of viable neurons and synapses (Neher et al., 2012; Koellhoffer et al., 2017; Liddelow et al., 2017; Morizawa et al., 2017; Soreq et al., 2017; Boisvert et al., 2018; Bussian et al., 2018; Clarke et al., 2018; Forloni and Balducci, 2018; Jung and Chung, 2018). In contrast, senolysis, elimination of aggressive glia, was associated with enhanced memory in animal models, suggesting a therapeutic strategy (Koellhoffer et al., 2017; Bussian et al., 2018; Forloni and Balducci, 2018).

The infection-senescence link cannot be considered without mentioning the role of iron, a biometal indispensable to both the host and invading pathogens. Iron is well-known for inducing DNA damage and senescence in many cell types, including the ECs, linking it to microbial brain entry (Mollet et al., 2016). The association of AD with iron dysmetabolism is well-documented as, aside from microbial survival, this biometal was linked to tau pathology, reactive oxygen species (ROS), and neuroinflammation (Nakamura et al., 2016; Masaldan et al., 2018; Rao and Adlard, 2018).

Finally, aside from the pathogenetic mechanisms, this article discusses potential AD targets and therapeutic strategies, including inflammasome inhibitors, iron chelators, senolytic antibiotics, mitophagy inducers, and epigenetic reprograming of metabolism.

## Beta Amyloid: Friend or Foe?

Amyloid cascade hypothesis, the stipulation that toxic β-amyloid oligomers and fibrils are the primary cause of AD, has been the leading paradigm that drove research in this neurodegenerative disorder for the past three decades. According to this model, β-amyloid induces the formation of NFTs, leading to neuronal and synaptic loss that ultimately impact the memory (Hardy and Higgins, 1992; Morris et al., 2014). Lately, new hypotheses have emerged as numerous anti-amyloid drugs and vaccines failed to improve cognition in clinical trials, and several studies pointed to inconsistencies in the amyloid paradigm (Table 1).

**Table 1:** Perceived inconsistencies in the amyloid cascade hypothesis emphasized by novel studies.

**Findings**	**References**
β-amyloid volume does not corelate well with the degree of memory loss.	Ingelsson et al., 2004; Huber et al., 2017; Kametani and Hasegawa, 2018
NFTs, neuronal loss and memory deficit have not been observed in BRI2-Aβ AD mouse model despite abundant β-amyloid deposits.	Kim et al., 2013
Neuroimaging studies visualized significant β-amyloid deposits in up to a third of elderly individuals with no memory loss.	Edison et al., 2007; Li et al., 2008; Rodrigue et al., 2013; Higashi et al., 2018
CNS infections were observed in some clinical trials after β-amyloid clearance.	Orgogozo et al., 2003; Alonso et al., 2018; Brothers et al., 2018

**Figure 1:**
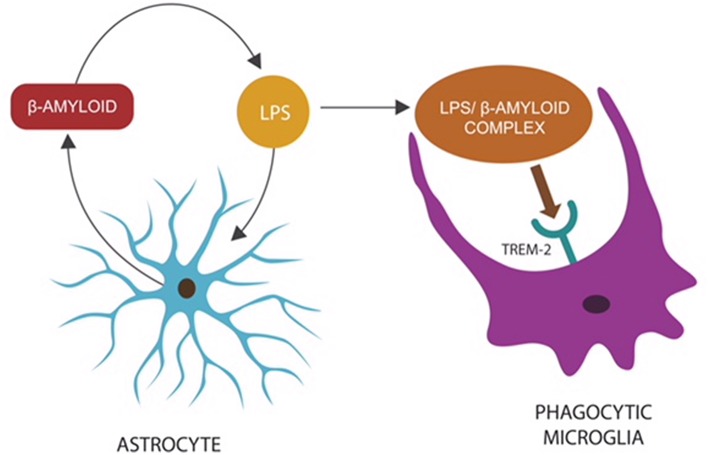
Microbes or LPS that access the CNS comprise “danger” signals, triggering an innate immune response: the release of β-amyloid by astrocytes to opsonize “the intruder,” preparing it for phagocytosis (Zhan et al., 2018). The LPS–β-amyloid complex is subsequently engulfed by microglia, eliminating the “danger.” Microglial TREM-2, a β-amyloid receptor, initiates phagocytosis by binding the entire complex (Zhao et al., 2018). This mechanism may explain the reason TREM-2 genetic variants (with loss of function) present with impaired phagocytosis and β-amyloid accumulation (Guerreiro et al., 2012; Zhan et al., 2018).

Recent studies have indicated that β-amyloid may function as an AMP released by the host innate immunity in response to invading pathogens (Spitzer et al., 2016; Fülöp et al., 2018a; Gosztyla et al., 2018). This is further supported by the observation that in CNS infections, microglia, and astrocytes secrete a multitude of AMP peptides demonstrated to augment host defenses (Ransohoff and Brown, 2012; Williams et al., 2012; Frost and Li, 2017). Moreover, β-amyloid, released by astrocytes and neurons, presents with antibacterial, fungicidal, and anti-herpes simplex virus, type 1 (HSV1) properties (Lukiw et al., 2010; Bourgade et al., 2016; Frost and Li, 2017; Eimer et al., 2018). This is significant since HSV1, an established disruptor of biological barriers, was found to play a major role in the etiology of both AD and intestinal pathology (Brun et al., 2018; Hogestyn et al., 2018; Itzhaki and Lathe, 2018). Interestingly, a novel study has reported that β-amyloid may work in tandem with a second AMP, probably to augment its microbicidal functions (De Lorenzi et al., 2017). Furthermore, under normal circumstances, β-amyloid may act as an opsonin, attaching to CNS microorganisms and/or their molecules to prepare them for microglial phagocytosis (Figure 1).

### Oral and Microbial Tolerance

Other microbial antigens, including bacterial amyloids (also known as curli fibers) and *P. gingivalis*-released gingipains, may trigger β-amyloid upregulation to opsonize these “danger” molecules (Tükel et al., 2009; Hill et al., 2014; Kumar et al., 2016).

Novel studies have shown that curli fibers derived from gut microbes play a major role in promoting immune tolerance to commensals as well as oral tolerance (immune unresponsiveness to antigens administered by mouth, including food) (Barnhart and Chapman, 2006; Oppong et al., 2015). These curli functions are protective when the microbes are confined to the GI tract but become detrimental after translocation as the systemic immunity (which is not subject to oral tolerance) is activated by curli. Indeed, curli fibers were demonstrated to activate or inhibit innate immune responses, depending on the portal of entry: systemic administration of curli augments, while oral ingestion lowers immune responses (Tursi and Tükel, 2018). As curli fibers promote oral tolerance, their administration by mouth was found to restore the integrity of intestinal barrier, suggesting a potential antitranslocation strategy (Tursi and Tükel, 2018). Indeed, a bioengineered curli was recently utilized as a restorative therapy for intestinal barrier (Duraj-Thatte et al., 2018). Furthermore, like curli, oral administration of LPS derived from *Bacteroides vulgatus* and *Bacteroides dorei* was demonstrated to promote tolerance by blocking rather than activating intestinal toll-like receptor 4 (TLR-4), pointing to a mechanism for tolerization (d'Hennezel et al., 2017). Interestingly, live *B. vulgatus* and *B. dorei* were recently investigated as therapy for coronary artery disease (CAD), another condition linked to the translocation of gut microbes (Yoshida et al., 2018).

Upon accessing the CNS, curli fibers likely trigger β-amyloid synthesis, an innate immune response, causing the accumulation of this peptide. Others have suggested that curli serve as templates for β-amyloid seeding, resulting in wider CNS depositions (Friedland and Chapman, 2017). We propose that bacterial amyloids are antigens that trigger a defensive response, β-amyloid overproduction, to eliminate “danger” signals.

Aging disrupts both oral and microbial tolerance, leading to immunogenicity and inflammation in response to commensals, disrupting the intestinal barrier, a portal for microbial dissemination (Kato et al., 2003; Santiago et al., 2011).

Taken together, commensal gut microbes live in symbiosis with the host for as long as they are confined to the GI tract where the local immune system maintains a tolerant environment. This symbiosis is dramatically altered upon microorganisms' translocation as gut microbes and their antigens activate systemic immunity. Aging alters both oral and microbial tolerance, disrupting intestinal barrier and enabling microbial translocation. Upon CNS entry, microbes and their molecules induce β-amyloid overproduction. In summary, microbial containment inside the gut lumen is a key objective in the prevention of neurodegeneration, including AD.

### Antimicrobial Peptides, Aging, and β-Amyloid

Over the past two decades, AD studies have focused primarily on the detrimental functions of β-amyloid, placing less emphasis on its physiological roles: protection against infections and cancer, BBB repair, and synaptic maintenance (Brothers et al., 2018). The presence of β-amyloid in various tissues and organs of older individuals and AD patients has gained a new significance in the light of this biomolecule functioning as an AMP (Joachim et al., 1989). For example, novel studies have detected microorganisms in older individuals' tissues, including the liver, skeletal muscles, and brain, suggesting that increased microbial burden triggers higher β-amyloid synthesis (Lluch et al., 2015). Furthermore, preclinical studies have reported age-related upregulation of AMPs in senescent tissues, implying that these defense peptides may be directly proportional to the bacterial load (Dinakaran et al., 2014). Interestingly, numerous studies over the past decade linked tissue pathogens to chronic illnesses, including CAD, cancer, stroke, type 2 diabetes mellitus (T2DM), and AD, likely implicating the translocated gut microbes in their etiology (Elkind et al., 2009; Dapito et al., 2012; Sato J. et al., 2014).

Other studies have found that the function of AMPs as antiviral and anticancer agents, suggesting that carcinogenesis and infection are handled in a similar fashion by the immune system (Hoskin and Ramamoorthy, 2007; Suttmann et al., 2008; Pandey et al., 2016). Interestingly, β-amyloid has been shown to display not only anti-HSV1 but also antimalignant properties, further suggesting an adaptive role for this peptide (Bourgade et al., 2015; Mizejewski, 2017).

AMPs have been linked to autophagy, a process involved not only in the clearance of damaged cells and molecules but also in antimicrobial defenses, as they are effective against facultative intracellular pathogens, like *P. gingivalis* (Muciño et al., 2016).

Another AMP, neuropeptide-like protein 29 (NLP-29), was found to promote the autophagy of damaged dendrites (dendrophagy) in *Caenorhabditis elegans*, extending the role of AMPs beyond infection and cancer (Lezi et al., 2018). Interestingly, fungi were shown to subvert NLP-29, inducing neuronal senescence, linking them to brain aging (Alonso et al., 2018). This is significant since fungal infections have previously been associated with AD and aging.

Other studies have reported the existence of antiretroviral AMPs, which, like antiretroviral drugs, interfere with the expression of retroviral genes, including Arc (Tencza et al., 1997; Nelson et al., 2003; Kriesel et al., 2017). Cognition-related neuronal gene Arc was demonstrated to migrate from neuron to neuron in a retroviral fashion, possibly linking antiretroviral drugs to cognition (Ashley et al., 2018; Pastuzyn et al., 2018). Interestingly, HSV1 was associated with altered transcription of Arc, linking this virus once again to neuronal senescence and memory loss (Penner et al., 2010; Bi et al., 2018; Acuña-Hinrichsen et al., 2019; Man et al., 2019). This finding is in line with novel epidemiological studies that have connected HSV1 to cellular senescence and AD (Dowd et al., 2017).

Finally, AMPs were found crucial for the integrity of intestinal barrier, suggesting their upregulation as a strategy against bacterial translocation (Robinson et al., 2015). Indeed, a recently synthesized AMP has been shown to neutralize LPS, indicating potential antitranslocation benefits (Li L. H. et al., 2017). In addition, lactoferrin, a recently identified AMP, was found protective of intestinal barrier (Hering et al., 2017).

### Senescence and Extracellular Vesicles

Most cells in the human body release extracellular vesicles (EVs) to mediate cellular crosstalk and the exchange of metabolites. Gram-negative microbes also signal with EVs (also called outer membrane vesicles) to facilitate immune evasion (Rodrigues et al., 2018). For example, *P. gingivalis* emits EVs that trigger pyroptosis in macrophages and microglia, effectively eliminating these key host defenses (Fleetwood et al., 2017). *P. gingivalis*-derived EVs have been demonstrated to contain antigens, including gingipains and fimbriae, known for disrupting ECs, causing BBB and intestinal barrier damage (Mantri et al., 2014).

Along these lines, novel studies show that the age-related gut microbial shift may be orchestrated via EVs released by microorganisms to alter local immunity and the intestinal barrier (Ahmadi Badi et al., 2017). Other studies have shown that under normal circumstances, the thymus gland releases EVs that act on gut-associated lymphoid tissue (GALT), promoting immunological tolerance to gut microbes (Skogberg et al., 2015). Age-related thymic involution may lower commensals' tolerance, engendering inflammation, and intestinal barrier disruption (Skogberg et al., 2015; Li P. et al., 2016). Interestingly, a recent preclinical study has shown that thymic EVs derived from young donors reversed the inflammaging in older recipients, suggesting that functional restoration of this gland may comprise a senotherapeutic strategy (Wang et al., 2018).

Recent studies have shown that senescent cells release more EVs than their younger counterparts, suggesting a mechanism for molecular waste disposal (Falsone and Falsone, 2015; Takasugi, 2018). For example, senescence-associated secretory phenotype (SASP) has been linked to the accumulation of cytosolic DNA in senescent cells, while DNA export via EVs was shown to inhibit this phenotype (Takahashi et al., 2018). These findings suggest that facilitation of DNA egress from senescent cells may comprise an effective senotherapeutic intervention. In this regard, the antibiotic ciprofloxacin was shown to facilitate DNA export from senescent cells, suggesting anti-SASP properties (Németh et al., 2017). Interestingly, malignant cells also display enhanced DNA export via EVs, suggesting that SASP may be associated with carcinogenesis (Rajagopal and Harikumar, 2018). Conversely, heparin was shown to block recipient cells' uptake of tumor and non-tumor-derived EVs, suggesting a potential strategy (Atai et al., 2013). Indeed, heparin was demonstrated to mimic extracellular DNA, probably interfering with SASP signaling (Jung et al., 2015; Mishra and Horswill, 2017).

### Aging and Biological Barriers in Alzheimer's Disease

Cellular senescence is a program of permanent replication arrest which, under normal circumstances, lowers the risk of carcinogenesis. Prompt removal of senescent cells by the immune system prevents their accumulation and the subsequent inflammation (Oppong et al., 2015). Aging has been shown to alter this process, engendering both inflammaging and immunosenescence (Olivieri et al., 2018).

More than five decades ago, Hayflick established that cells divide a limited number of times after which they undergo replicative senescence and apoptosis (Hayflick and Moorhead, 1961). Later on, it was established that the senescence program can be activated prematurely by numerous endogenous or exogenous toxins, including the microbes and their antigens, such as LPS (Nakamura et al., 2016; Calvani et al., 2018; Kritsilis et al., 2018).

Compelling evidence indicates that cellular senescence contributes to organismal aging and the risk of developing age-related diseases, including AD (Jeyapalan and Sedivy, 2008). A growing number of studies have demonstrated that senescent cells' SASP secretome can activate the senescence program in healthy cells, propagating this phenotype throughout the surrounding tissues (Nelson et al., 2012). Conversely, senolysis, senescent cell removal, has been shown to restore homeostasis, ameliorating age-related symptoms (Baar et al., 2017; Kirkland et al., 2017). Accumulation of senescent cells and SASP-derived molecules, due to overproduction or impaired clearance, comprises an early sign of AD (Boccardi et al., 2015; Childs et al., 2015; Kritsilis et al., 2018).

Histologically, senescent cells are enlarged, presenting with β-galactosidase and lipofuscin aggregates. Functionally, they are resistant to apoptosis and metabolically active as evidenced by the intact mammalian target of rapamycin (mTOR) and the SASP secretome. Since senescent cells continue to express mTOR, targeting this molecule may comprise a senotherapeutic strategy for SASP inhibition (Walters and Cox, 2018). For example, rapamycin, an mTOR inhibitor and a natural macrolide antibiotic, was shown to block both cellular senescence and SASP (Wang R. et al., 2017; Wang S. et al., 2017). In addition, as mTOR signaling also modulates ECs synthesis of nitric oxide (NO), a trophic molecule for endothelia, targeting mTOR may restore the integrity of biological barriers, including the BBB (Cheng et al., 2008; Van Skike and Galvan, 2018). Interestingly, *P. gingivalis* was found to alter mTOR signaling, linking this microbe once again to EC senescence (Stafford et al., 2013). Conversely, azithromycin, an anti-*P. gingivalis* macrolide antibiotic and mTOR modulator, was found to have senotherapeutic properties, indicating potential benefits in AD (Maezono et al., 2011; Ratzinger et al., 2014; Ozsvari et al., 2018; Weng et al., 2019).

### The Senescent Intestinal Barrier

**Figure 2:**
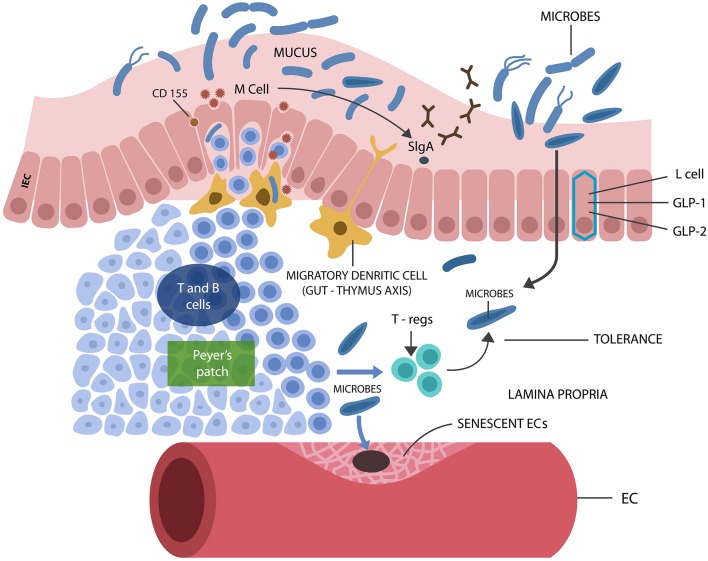
M cells interact with luminal microbes, introducing microbial antigens to the T and B cells, engendering tolerance via Tregs and IL-10 secretion. M cells promote the expression of the mucosal protector, SIgA. L cells synthesize IECs protecting biomolecules GLP-1 and GLP-2. Senescent ECs cause endothelial disruption, allowing pathogens into the circulatory system, from where they find their way into the CNS.

The gut microbial community, consisting of bacteria, fungi, archaea, viruses, and protozoans, live in symbiosis with the human host, contributing to metabolism and immune homeostasis in exchange for nutrients and habitat (Jandhyala et al., 2015). Intestinal epithelial cells (IECs), a one cell layer, separate the host from trillions of microbes and antigens, preventing their translocation outside of the GI tract where systemic immunity is intolerant of them. Aside from IECs, GALT (the GI tract immune system) contributes to the integrity of the intestinal barrier by blocking immunogenicity to beneficial microorganisms, ensuring their containment in the GI tract (Hwang et al., 2012). Loss of tolerance to gut commensals was shown to cause immune activation, barrier disruption, and translocation (Ramanan and Cadwell, 2016). GALT facilitates microbial tolerance by promoting the differentiation of IL-10 secreting B and regulatory T cells (Tregs) (Kelsall and Leon, 2005) (Figure 2). In addition, GI tract lactobacilli, bifidobacteria, and *Bacteroides* also facilitate microbial acceptance as they promote oral and microbial tolerance (Cebula et al., 2013; Kayama and Takeda, 2014; Nakamoto et al., 2017). Tolerance is believed to be initiated during the early development when GALT receives thymic input, generating a long-lived population of T cells that facilitate microbial tolerance even after the involution of this gland (Cebula et al., 2013).

Maintenance of a microbe-friendly GI tract milieu, completely isolated from the systemic immunity, is crucial for averting microbial translocation, a phenomenon that may initiate age-related diseases, including AD. Conversely, restoring the integrity of biological barriers and limiting microbial translocation should be the primary objective of senotherapy. Indeed, recent studies have associated mTOR inhibition with the restoration of intestinal barrier damaged by *P. gingivalis* (Xu et al., 2015; Nakamoto et al., 2017; Ji et al., 2018; Kato et al., 2018). Inhibitors of mTOR receptors were shown to lower GI tract immunogenicity as a bilateral regulation exists between gut microbes and mTOR, in which the later regulates microbial composition, while the former modulates mTOR expression (Salem et al., 2018). This is significant as it demonstrates that mTOR manipulation may reverse the age-related shift in gut microbiota, lowering the preponderance of pathogenic species and preserving intestinal barrier. On the other hand, microbiota manipulation, for example, via the fecal transplant, may reverse the preponderance of gut LPS-generating species in favor of beneficial microbes, such as *Bacteroides*, protecting intestinal barrier (Nagpal et al., 2018). Along these lines, a large epidemiological study found that *Bacteroides* species were less represented in the GI tract of AD patients compared to other microbes, indicating that microbiota manipulation may preempt neurodegeneration (Saji et al., 2019).

Age-related microorganismal shift toward Gram-negative bacteria and LPS induces EC senescence and apoptosis and IEC and GALT damage, disrupting the intestinal barrier (Hoyt et al., 1996; Richter et al., 2012; Nagele et al., 2013; Ke et al., 2018; Sanada et al., 2018; Hou et al., 2019). In addition, several gut microbes were demonstrated to upregulate the host tumor necrotic factor alpha (TNF-α) and interferon-gamma (IFN-γ), increasing intestinal permeability (Al-Sadi and Ma, 2007). Furthermore, these cytokines activate NLRP3, generating IL-1β, a known BBB disruptor (Wang et al., 2014).

### Does Aging Start in the Gut?

It was recently reported that GALT dysfunction may occur prior to the systemic immune deterioration, suggesting that immune aging and, perhaps, aging in general could originate in the GI tract with the loss of tolerance to commensals and barrier disruption (Koga et al., 2000; Sato S. et al., 2014).

It was recently demonstrated that glucagon-like peptide-1 (GLP-1) secreted by the gut enteroendocrine (L) cells binds to its receptor, GLP-1R, facilitating immunological tolerance (Yusta et al., 2015). Others have shown that under normal circumstances, LPS upregulates GLP-1, suggesting that this hormone may display AMP-like characteristics (Lebrun et al., 2017). Interestingly, GLP-1R agonists were recently demonstrated to block the conversion of trophic into A1 astrocytes, linking this peptide to CNS homeostasis (Yun et al., 2018). GLP-1R agonists, established therapeutics for T2DM, were previously shown to protect cognition; thus, liraglutide and exenatide are currently in clinical trials for AD and Parkinson's disease (PD), respectively (Kim et al., 2017; Batista et al., 2018; Cummings et al., 2018).

Aside from GLP-1 secretion, L cells sense pathogen-derived molecules, likely suggesting that GLP-1 functions as an AMP (Greiner and Bäckhed, 2016; Lebrun et al., 2017). Aging has been associated with decreased number of L cells, accounting for the loss of GI tract immunological tolerance (Drozdowski and Thomson, 2006; Wu et al., 2018). Aside from L cells, GALT dysfunction may be linked to the loss of membranous (M) cells, known for producing secretory immunoglobulin A (SIgA), an IEC immune protector (Mantis et al., 2011; Kobayashi et al., 2013; Sato S. et al., 2014; Ohno, 2016). Furthermore, aging has been associated with the loss of LPS-binding protein (LBP), another possible AMP, known for its trophic effects on the intestinal barrier (Schmucker et al., 2003; Hamann et al., 2005; Richter et al., 2012).

Another mechanism responsible for tolerance to commensal flora may involve the CD155 poliovirus receptor, expressed by M cells. CD155 binds to T cell co-inhibitory receptor TIGIT (T cell Ig and ITIM domain), initiating the release of IL-10 (Lozano et al., 2013; Ohno, 2016). Dysfunctional TIGIT has been associated with T cell senescence, linking immune aging to the GI tract (Solomon and Garrido-Laguna, 2018). On the other hand, TIGIT blockade, a well-known cancer treatment, activates immunity (Song et al., 2018). This is significant, since T cell co-inhibitory receptors are routinely hijacked by pathogens to lower host immunity and evade detection (Attanasio and Wherry, 2016). For example, *P. gingivalis* is known for subverting programmed death-1 (PD-1), a co-inhibitory receptor, to escape host immunity (Groeger et al., 2017).

### The Senescent Blood-Brain Barrier

ECs pave the interior wall of blood vessels and capillaries, contributing to blood flow, platelet function, and immunity (Ross, 2018). Microorganisms use the host circulatory system to travel around the body, crossing the ECs to enter and exit the bloodstream (Lubkin and Torres, 2016). To facilitate this process, pathogens trigger EC senescence and apoptosis, disrupting biological barriers, including the BBB (Kim, 2008). This action is counteracted by the ECs' secretion of NO, an endothelial protector (Hayashi et al., 2008; Austin et al., 2013). Decreased NO generation was associated with NLRP3 inflammasome activation, aging, and AD (Mao et al., 2013; Sverdlov et al., 2014).

Astrocytic end-feet, ECs, and pericytes comprise the BBB or neurovascular unit (NVU), which feeds neuronal networks, enabling their function (Filosa et al., 2015; Tarantini et al., 2016). Several studies have shown that BBB disruption is an early AD marker, indicating a potential portal for microbial entry into the CNS (Montagne et al., 2015; Nation et al., 2019). A novel study measured platelet-derived growth factor receptor-beta (PDGFRβ), a pericyte marker, and showed that its deficit increased the permeability of BBB, contributing to AD (Nation et al., 2019). In addition, recent AD postmortem studies have associated loss of pericytes with BBB dysfunction in various cortical areas, including the hippocampus (Miners et al., 2017; Schultz et al., 2018).

Pericytes have been reported to play a major role in CNS antimicrobial defenses by secreting microbicidal molecules, including IL-1β, IL-6, and TNF-α (Alcendor et al., 2012; Hurtado-Alvarado et al., 2014; Stark et al., 2018). Several pathogens were demonstrated to evade host immunity by subverting the pericytes, linking these cells to microbes and their portal of entry (Alcendor et al., 2012). For example, a new study has demonstrated that heme-dependent pathogens can damage ECs and pericytes to extract this iron protein from the circulating red blood cells (Choby and Skaar, 2016; Erdei et al., 2018). Along these lines, to acquire heme, *P. gingivalis* releases gingipain, which attaches to the EC receptor E-selectin, disrupting these cells (Komatsu et al., 2012; Smalley and Olczak, 2017). Other studies have reported that fimbriae, another *P. gingivalis* antigen, binds EC-expressed complement receptor 3, inducing immune tolerance to enter the CNS undetected (Hajishengallis et al., 2008). This is significant because upregulated complement component C1q and its downstream molecule C3 were linked to AD via A1 astrocytes induction (Wu et al., 2016; Liddelow et al., 2017; Morgan, 2017). ECs are extremely susceptible to microbial disruption as they express the tolerance-inducing complement pathway genes; therefore, when pathogens subvert these cells, they trigger immune unresponsiveness (Walker et al., 2007; Shi et al., 2017).

Aside from *P. gingivalis, Helicobacter pylori* and *Escherichia coli* were found to induce EC senescence and apoptosis, linking them to the disruption of biological barriers (Munshi et al., 2002; Krishnan et al., 2012). Moreover, HSV1, connected to both atherosclerosis and AD, was demonstrated to invade ECs, activating glycogen synthase kinase 3 beta (GSK3β), an enzyme previously associated with neurodegeneration (Key et al., 1990; Piacentini et al., 2015; Rybakowski, 2019). Interestingly, lithium, a GSK3β blocker, also presents with anti-HSV1 properties, suggesting a protective effect on endothelia (Amsterdam et al., 1990; Bosche et al., 2016). Indeed, the beneficial effect of lithium in AD may involve endothelial restoration (Bosche et al., 2016; Cummings et al., 2018).

Immune aging or immunosenescence promotion, engendering immune failure, is another mechanism utilized by gut microbes to avoid detection and access the CNS unopposed (Blazkova et al., 2009; Alvarez-Arellano and Maldonado-Bernal, 2014; Aguilera et al., 2018; Costantini et al., 2018).

Taken together, pathogen-induced pericyte and EC senescence and apoptosis along with impaired immune function facilitate microbial translocation into the brain with subsequent AD pathology.

### Senescence and Inflammasomes

**Figure 3:**
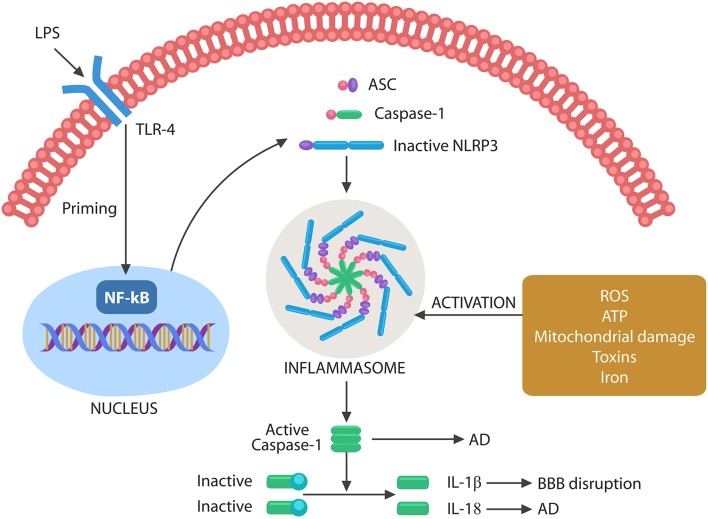
Schematic representation of NLRP3 activation by microbes or their molecules. Microbes or LPS binds to TLR-4, activating the nuclear NF-kB that primes NLRP3. The second step necessary for NLRP3 activation can be composed of various exogenous or endogenous stimuli, including ROS, ATP, DNA, defective mitochondria, iron, and toxins. Assembled inflammasome activates caspase-1, which in turn cleaves immature IL-1β and IL-18 into their active forms. Caspase-1 and IL-18 have been involved in AD pathogenesis, while IL-1β disrupts the BBB, facilitating brain translocation of gut microbes.

It has been well-established that inflammation and cellular senescence are closely related, but the role of pathogens in this process has been less emphasized (Balistreri et al., 2013; Secher et al., 2013; Lewinska and Wnuk, 2017; Rybakowski, 2019). At the molecular level, cellular senescence is believed to be initiated by the nuclear translocation of the NF-kB transcription factor, a molecular event that primes NLRP3 inflammasomes (McCool and Miyamoto, 2012; Birch and Passos, 2017) (Figure 3).

Several antibiotics, including minocycline and macrolides, present with both antimicrobial and anti-inflammatory properties as they de-escalate NLRP3 (Pradhan et al., 2016). Recent studies have shown that minocycline also presents with senolytic properties as aside from inhibiting NLRP3, it facilitates senescent cell removal (Labro, 2002; Li J. et al., 2016; Lee et al., 2017).

Other antibiotics with senotherapeutic actions include azithromycin and rifampicin, suggesting that infection, inflammation, and cellular senescence are related phenomena (Golegaonkar et al., 2015; Lendermon et al., 2017; Ozsvari et al., 2018).

Inflammasomes are macromolecular complexes that sense pathogen-associated molecular patterns (PAMPs) or damage-associated molecular patterns (DAMPs) via cytosolic NLRP3 composed of NOD-like receptors, adapter proteins, apoptotic speck containing molecules with a CARD (ASC), and pro-caspase-1 (Uekawa et al., 2004; Broz and Monack, 2011; Wang et al., 2014; Schetters et al., 2018).

Inflammasome assembly requires two steps, a priming event, triggered by NF-kB nuclear translocation, and an activating step, induced by toxins, iron, mitochondrial damage, cytosolic DNA, extracellular ATP, or ROS. Inflammasome assembly activates caspase-1, which in turn cleaves pro-IL-1β and pro-IL-18 into their mature forms (Figure 3). These cytokines have been involved in both BBB disruption and AD (Heneka et al., 2013; Freeman and Ting, 2016; Malik and Kanneganti, 2017).

Pyroptosis is a programmed cell death triggered by infection-induced NLRP3 activation mediated by caspase-1,−4, and−5. Caspase-1 is the product of NLRP3 assembly, while caspase-4 and−5 are LPS activated (Man et al., 2017). Caspases perforate cell membranes via gasdermin D, a pore-forming protein, spilling intracellular content into the ECS, a process that triggers inflammation (Ma et al., 2018). Pyroptosis has been documented in the pathogenesis of neurodegenerative disorders (Walsh et al., 2014; Wang et al., 2014; Ma et al., 2018). In fact, it is believed that pyroptosis and not apoptosis leads to neuronal loss in AD (Bai, 2018; Fali et al., 2018). Moreover, recent studies have reported NLRP3-induced pyroptosis in ECs, likely explaining the disruption of intestinal barrier and BBB during the aging process (Lei et al., 2018; Zhaolin et al., 2018).

Mitochondrial damage was recently recognized as a key NLRP3 activator, emphasizing the role of these organelles in both aging and AD. For example, mitochondrial dysfunction-associated senescence (MiDAS) is an aging phenotype with a specific secretome which, like SASP, can propagate cellular aging throughout the tissues (Gallage and Gil, 2016). Other studies have shown that mitochondrial content, especially mtDNA, in contact with the cytosol activates cellular senescence and SASP, while cytosolic DNA removal inhibits both (Takahashi et al., 2018; Takasugi, 2018). *P. gingivalis*-induced cellular senescence may involve mtDNA as this microbe has been known for inflicting mitochondrial damage (Bullon et al., 2011). This is in line with a novel study that identified cell free-DNA (cfDNA) as an aging and AD marker, suggesting that cytosolic DNA exported into the ECS may suppress SASP (Takousis et al., 2018; Teo et al., 2018). Together, these studies suggest that enhancing the clearance of cytosolic DNA may facilitate senolysis, lowering the senescent cell burden in tissues and organs (Takousis et al., 2018; Teo et al., 2018).

### Senescent Neuron and the Cell Cycle

Accumulating evidence indicates that aging neurons activate a special senescence program, defined as senescence after differentiation (SAD), a phenotype marked by upregulation of β-galactosidase, lipofuscin, SASP, and IL-6 (Jurk et al., 2012; Naylor et al., 2012; Tan et al., 2014). Along these lines, a new study identified senescent neurons in the orexinergic, cholinergic, and dopaminergic tracts of the brainstem and basal forebrain, probably indicating that some neuronal populations undergo senescence earlier than others (Panossian et al., 2011). As opposed to senescent somatic cells which arrest proliferation irreversibly, old neurons may do the opposite, reenter the cell cycle, triggering their own demise (Paquola et al., 2016; McConnell et al., 2017; Verheijen et al., 2018). Indeed, the expression of neuronal cell cycle proteins was detected in both healthy seniors and AD patients, suggesting that these molecules are senescence associated (van Leeuwen and Hoozemans, 2015; Frade and López-Sánchez, 2017). In addition, a novel AD postmortem study has linked neuronal senescence with both aggregated tau and neuronal cell cycle reentry, identifying both as age-related traits (Musi et al., 2018). Moreover, aberrant neuronal cell cycle reentry has been associated with a senescence-linked protein, cyclin-dependent kinase 5 (Cdk5), which phosphorylates both tau and the retinoblastoma protein (pRb) (Mao and Hinds, 2010). The nuclear localization of Cdk5 was found necessary for maintaining neuronal cells in post-mitotic state (Zhang et al., 2008). Conversely, egress of this protein from the nucleus activates the cell cycle (Hamdane et al., 2005; Crews et al., 2011; Hradek et al., 2015; Na et al., 2015). This may be the case in AD, in which microbes and/or LPS may trigger Cdk5 nuclear export and neuronal cell cycle activation (Zhang et al., 2016). In PD animal models, neuronal Cdk5 was found to activate NLRP3, initiating the cell cycle (D'Angelo et al., 2017; Bai, 2018; Wilkaniec et al., 2018). Indeed, to interact with the cytosolic NLRP3, Cdk5 must exit the nucleus, enabling this phenomenon. Conversely, LPS removal via LBP probably promotes Cdk5 nuclear reentry, stabilizing neuronal cells in post-mitotic state (Pretorius et al., 2018).

Iron is a well-known activator of neuronal cell cycle, probably due to DNA damage and NF-kB/NLRP3 activation, suggesting that iron chelators may have senotherapeutic properties (Nakamura et al., 2016; Ashraf et al., 2018; Manickam et al., 2018) (discussed in “Senescence and Iron” section).

It is currently believed that senescent post-mitotic cells, including the neurons, reenter the cell cycle to trigger their own demise. This is thought to take place as these cells lack the molecular machinery to complete mitosis, activating death programs instead (Kruman et al., 2004). For example, in muscular degeneration, adult post-mitotic myocytes were shown to reengage the cell cycle, triggering their own death (Sharma et al., 2017). Conversely, cell cycle inhibitors were recently found neuroprotective in AD organoid models, suggesting a possible therapeutic strategy (Hor et al., 2018).

Most recent studies have suggested that some neuronal populations reentering the cell cycle do not always undergo cell death but remain in the aneuploid state for the rest of their lives (Frade and López-Sánchez, 2017). For example, loss of the p53 tumor suppressor, a DNA repair protein, was associated with neuronal survival in the aneuploid state (Barrio-Alonso et al., 2018).

Neuronal cells have recently been reported to present with variable DNA content from one cell to another (somatic mosaicism), especially during the early development and old age (Paquola et al., 2016; McConnell et al., 2017; Sharma et al., 2017; Caneus et al., 2018; Leija-Salazar et al., 2018; Verheijen et al., 2018; Villela et al., 2018). This finding led to the development of a new field, defined as the brain somatic mosaicism (Paquola et al., 2016; McConnell et al., 2017). We speculate that this phenomenon is the result of senescent neurons reengaging the cell cycle and surviving in aneuploid states. In AD, neuronal somatic mosaicism may be reflected in the aneuploidy-induced APP gene variants (Bushman et al., 2015). Interestingly, a recent study has suggested that APP variants are generated via RNA retro-insertion into the DNA, suggesting that antiretroviral drugs may be beneficial for AD (Lee et al., 2018). Others have argued that patients with HIV-associated neurocognitive disorders (HANDs) rarely experience improved memory while in treatment with antiretrovirals (McArthur et al., 2010; Vance et al., 2013). These contradictory findings indicate that more studies are needed to clarify the role of these agents in AD.

Finally, two questions beg for answers: Are aneuploid neurons viable and does it make sense to facilitate their survival?

Novel studies in regenerative medicine reported that facilitating cell cycle completion in senescent cardiomyocytes prevented their apoptosis (Anversa and Leri, 2013; Hesse et al., 2017; Locatelli et al., 2018). Helping neurons survive the cell cycle engagement may comprise a therapeutic strategy in AD, but only if aneuploid cells are functional (Frade and López-Sánchez, 2017). Conversely, preventing neurons from engaging the cell cycle, a more straightforward approach, may be accomplished by blocking the nuclear export of Cdk5 or suppressing this protein in the cytosol with Cdk5 blockers or lithium (Zhang et al., 2008; Carvalho et al., 2013).

### Senescent Astrocytes and Microglia

Astrocytes are the most numerous brain cells and their end-feet, ECs and pericytes comprise the BBB. Recent studies report that astrocytes are innate immune cells that, along with microglia, play a key role in the phagocytic removal of molecular waste, dead, or dying cells (Farina et al., 2007; Ransohoff and Brown, 2012; Morizawa et al., 2017). In addition, astrocytes generate AMPs, including β-amyloid, that may opsonize pathogens, facilitating their removal (Figure 1).

Preclinical studies have reported that astrocytes undergo both replicative and stress-induced senescence characterized by SASP, p16INK4a, and p21CIP1 markers; however, the difference between senescent and reactive astrocytes is not entirely clear at this time (Hou et al., 2018; Kritsilis et al., 2018; Maciel-Barón et al., 2018; Perez-Nievas and Serrano-Pozo, 2018). Recent studies seem to indicate that these phenotypes may be closely related or even identical as upregulated inflammatory and synapse-eliminating genes were found in both senescent and reactive astrocytes (Crowe et al., 2016; Boisvert et al., 2018). Along these lines, the aggressive A1 astrocytes may be senescent as they also upregulate inflammatory genes and eliminate healthy synapses (Liddelow et al., 2017; Morizawa et al., 2017; Clarke et al., 2018; Vilalta and Brown, 2018). In support of this hypothesis comes the recent finding that senescence-upregulated cytokines, TNF-α and IL-1, induce the A1 phenotype (Cartier et al., 2014; Altieri et al., 2017; Liddelow et al., 2017; Li P. et al., 2017; Yun et al., 2018).

Microglia are CNS innate immune cells, which, like macrophages at the body periphery, are vigilant and motile, characteristics that help them scrutinize the brain parenchyma, searching for “danger signals.” Microglia respond to invading pathogens by releasing pro-inflammatory cytokines which can trigger astrocytic senescence and reactivity (Cartier et al., 2014). In addition, under normal circumstances, microglia engulf senescent or dead cells, preventing their accumulation and the subsequent inflammation (Jung and Chung, 2018). Aging and immunosenescence were shown to alter microglial phagocytic function, generating inflammaging triggered by the accumulation of molecular waste and cellular corpses (Neumann et al., 2009; Koellhoffer et al., 2017).

Dystrophic microglia with growth arrest and senescent markers have been demonstrated in AD patients, but the difference between the reactive and dystrophic phenotype is unclear at this time (Flanary et al., 2007; Mosher and Wyss-Coray, 2014). Several studies have reported that although senescent microglia may lose their neuroprotective functions, their ability to mount inflammatory responses is preserved and even enhanced (Sierra et al., 2007; Davies et al., 2017). For example, senescent microglia have been shown to upregulate their TLRs, triggering exaggerated inflammation in response to minimal LPS stimulation. On the other hand, continuous LPS presence in the microglial environment induces immunosenescence with deficient phagocytosis (Yu et al., 2012). Recently, “dark,” hypervigilant microglia have been reported, likely representing senescent cells with aberrant phagocytic function (Bisht et al., 2016). Indeed, several studies report that in the presence of LPS, senescent microglia and astrocytes became neurotoxic, engaging in the phagocytosis of healthy neurons and synapses (von Bernhardi et al., 2015; Lana et al., 2017). Moreover, preclinical studies have shown that LPS-exposed microglia promote extracellular trafficking of hyperphosphorylated tau, a phenomenon inhibited by IL-10 (Liu et al., 2016; Magalhães et al., 2017; Hopp et al., 2018; Kametani and Hasegawa, 2018). Furthermore, microglial NLRP3 and its end products, IL-18, caspase-1, and IL-1β, have been associated with cellular senescence and AD (Griffin et al., 2006; Ojala et al., 2009; Cabral and de Lima, 2017). Conversely, caspase-1 inhibition ameliorates AD symptoms in animal models, suggesting a novel target (Yu et al., 2009; Cabral and de Lima, 2017; Flores et al., 2018).

Taken together, senescent microglia, incapable of proper immunosurveillance and phagocytosis, contribute to the accumulation of molecular waste, dead or dying cells, inducing inflammaging and immunosenescence. Astrocytes may respond to these microenvironmental changes by converting to the A1 phenotype marked by aberrant elimination of healthy synapses and neurons, a possible pathogenetic mechanism of AD.

## Senescence and Aerobic Glycolysis: Got Lactate?

In the nineteenth century, Otto Warburg noticed that cancer cells converted glucose to lactate even in the presence of oxygen, a metabolic modality defined as aerobic glycolysis (AG). Compared with healthy cells, which oxidize glucose in the mitochondrion via oxidative phosphorylation (OXPHOS), cancer cells prefer cytosolic AG that generates excessive amounts of lactate (Potter et al., 2016). These observations beg the question: Why do cancer cells need lactate?

Recent findings helped solve this dilemma by revealing that cancer, like microorganisms, escapes detection by reprograming host immune cells to AG, a metabolic modality associated with immune tolerance (Roland et al., 2014; San-Millán and Brooks, 2016). In addition, lactate generates an acidic microenvironment which inhibits the host immune system (Romero-Garcia et al., 2016). Furthermore, lactate upregulates snail, a tumorigenic protein (encoded by the SNAI2 gene) which inhibits host cellular senescence, a key antitumor defense (Li X. et al., 2018).

Novel studies found that AG is the metabolic preference not only of cancer cells but also of many healthy tissues, including the brain (Demetrius et al., 2015; Yellen, 2018). Under normal circumstances, 10–12% of brain glucose is catabolized via AG despite oxygen availability (Goyal et al., 2017). Furthermore, the brain regions most dependent on AG are those involved in rapid activation and information processing, such as cognition, memory, and alertness (Dienel and Cruz, 2016).

Along similar lines, it was recently reported that immune cells and ECs preferentially utilize AG, especially when exposed to LPS or pathogens, suggesting that for rapidly proliferating cells, the slower OXPHOS may be an inadequate energy modality (Jones and Bianchi, 2015; Boitsova et al., 2018; Escoll and Buchrieser, 2018; Liu R. et al., 2018; Salmond, 2018). On the other hand, senescent cells rely almost exclusively on OXPHOS, indicating that loss of AG is an aging biomarker (Wen et al., 2012; Li et al., 2013; Goyal et al., 2017). The molecular mechanism of age-related AG loss is incompletely understood; however, under normal circumstances, lactate is synthesized by astrocytes, a neurotrophic function that may be lost in senescent cells (Riske et al., 2016). It has been established that lactate interacts with its receptor GPR81 (also called HCAR1) to generate rapid ATP surges required for neuronal activation (Bergersen and Gjedde, 2012; Díaz-García et al., 2017). Unlike AG, OXPHOS may be incapable of supplying the neurons with large amounts of energy on short notice (Díaz-García et al., 2017).

Aside from the CNS, lactate-GPR81 signaling plays a key role in the GI tract, where it maintains the integrity of intestinal barrier by positively regulating IL-10 (Ranganathan et al., 2018). Aging alters the lactate-GPR81 axis, disrupting both commensals tolerance and the intestinal barrier. In addition, age-related loss of gut *Lactobacillus* species, a major source of intestinal lactate, may impair GPR81 signaling, increasing intestinal permeability and facilitating microbial translocation (Walter, 2008). Moreover, as lactate-GPR81 interaction blocks the NLRP3 activation, agonists at these receptors may present with senotherapeutic properties (Hoque et al., 2014; Errea et al., 2016; Nolt et al., 2018).

In AD, due to compromised lactate-GPR81 signaling, AG may be unavailable, rendering neuronal cells totally dependent on mitochondrial OXPHOS. However, as the aging process also impairs mitochondria, OXPHOS becomes unreliable, triggering an energy crisis (Fong et al., 2016). Furthermore, the compensatory mechanisms, including mitochondrial fission, fusion, and mitophagy, are also compromised in AD, further lowering OXPHOS and deepening the crisis (Santos et al., 2010; Fang et al., 2016; Kerr et al., 2017).

### Immunosenescence and Inflammaging

Immune system aging is closely linked to gut microbes and the loss of AG. Aging affects both the innate and adaptive immunity, but some cells are more affected than others (Burton and Stolzing, 2018). For example, AG-relying effector T cells are more impacted by age than the OXPHOS-preferring memory T cells (Carlos et al., 2018). As a result, antigens are remembered in old age, but they may trigger poor immune activation as evidenced by older individuals' weak response to vaccines (Lord, 2013).

Age-related immune alterations are captured by two words, inflammaging, denoting excessive innate immune activation, and immunosenescence, referring to the depletion of adaptive immune cells (Ventura et al., 2017; Fülöp et al., 2018a). The innate immune changes affect macrophages and natural killer (NK) cells at the body periphery as well as microglia and astrocytes in the CNS (Solana et al., 2018).

The NF-kB/NLRP3 axis was shown to regulate immune aging via proinflammatory cytokines IL-6, TNF-α, IL-1β, and IL-18 (Heneka et al., 2013; Couturier et al., 2016; Rea et al., 2018). Moreover, peripheral infections and inflammation were linked to microglial senescence, suggesting that interventions at the body periphery may influence central immunity (Netea and van der Meer, 2017; Cao and Zheng, 2018; Wendeln et al., 2018). Furthermore, infection with various pathogens, including cytomegalovirus (CMV), human immunodeficiency virus (HIV), HSV1, and *Toxoplasma gondii*, was implicated in immunosenescence and inflammaging, connecting these phenomena to microbes and their molecules (Solana et al., 2018). This is in line with the immune risk phenotype (IRP), a morbidity marker described in the elderly with CMV infection (Olsson et al., 2000).

Immunosenescence, marked by the depletion of adaptive immune cells, reflects thymic involution, a process starting in childhood and progressing at a rate of 3% per year throughout the adult life (Gui et al., 2012). The gradual loss of thymic function is manifested by a decrease in naïve T cells, increased number of memory cells, and downregulation of T cell receptors (TCRs) (Deleidi et al., 2015). Novel preclinical studies have linked thymic involution to the activation of the NF-κB/NLRP3 axis, while caspase-1 inhibitors were shown to restore thymic lymphopoiesis in elderly (Youm et al., 2012; Wen et al., 2018). Interestingly, viruses, bacteria, fungi, and parasites were demonstrated to infect the thymus directly, probably inducing senescence and premature atrophy (Nunes-Alves et al., 2013). This is significant as it links thymic involution to the loss of intestinal Tregs, impaired barrier function, and microbial translocation. Indeed, a thymus-gut axis was described during the early development when dendritic cells migrate from the GI tract to “educate” the thymus in commensals tolerance (Lathrop et al., 2011; Jain and Seed, 2016) Figure 2 (also discussed in “The senescent intestinal barrier”).

Age-related thymic involution was also associated with the loss of gut IL-10-secreting B cells, which, like Tregs, contribute to the microbial immune tolerance (Ghosh et al., 2013; van der Geest et al., 2016; Ip et al., 2017). Conversely, restoration of thymic function in older individuals or hormonal replacement may reverse immunosenescence, suggesting a novel therapeutic strategy. Indeed, preclinical studies reported that administration of EVs loaded with thymosin alpha 1 and melatonin restored the thymic function in older animals (Molinero et al., 2000; King and Tuthill, 2016; Wang et al., 2018).

### Senescence and Iron

**Figure 4:**
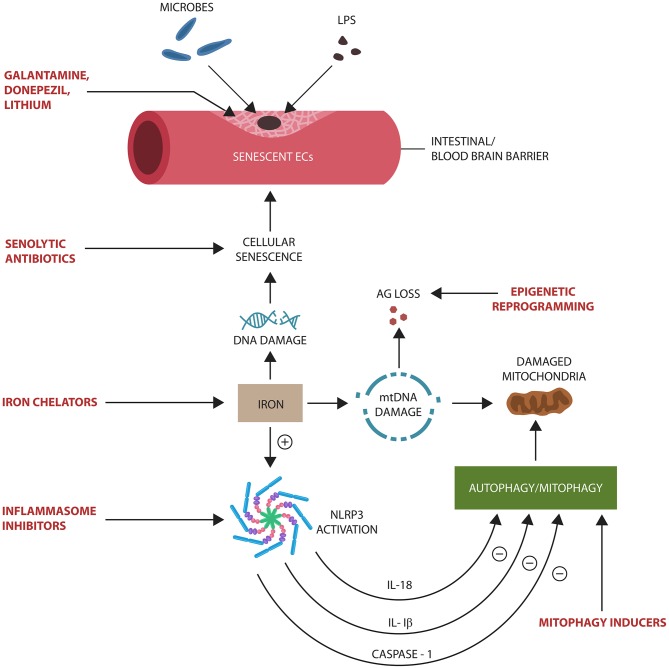
Potential senotherapeutic interventions in AD and the steps at which they may operate. NLRP3 end products, IL-1β, IL-18, and caspase-1, inhibit autophagy and mitophagy, contributing to the accumulation of senescent cells and damaged mitochondria. These, in turn, alter biological barriers, enabling microbial translocation and AG loss. Excess iron induces DNA and mtDNA damage, activating NLRP3 with subsequent cellular senescence.

Age-related iron dysmetabolism, a phenomenon well-documented in AD, is closely connected to cellular senescence and the loss of AG (Kelleher and Soiza, 2013; Ward et al., 2014; Lane et al., 2015). Iron is known for inducing DNA damage and EC senescence that increases BBB permeability and the risk of microbial translocation (Won et al., 2011; Mollet et al., 2016) (Figure 4). Moreover, iron was demonstrated to activate NLRP3 inflammasomes, linking this biometal to inflammation, dysfunctional mitochondria, and impaired mitophagy (Allen et al., 2013; Xiong et al., 2014; Nakamura et al., 2016). A component of iron–sulfur clusters (ISCs) and heme, iron has been demonstrated to alter mitochondrial glucose metabolism in response to pathogens (Horowitz and Greenamyre, 2010). For example, to deny microbes the access to glucose during infections, heme binds TLR-4, inducing hypoglycemia (Figueiredo et al., 2007; Weis et al., 2017). In AD however, hypoglycemia may be a double-edged sword as it may deepen the cellular energy crisis (Fong et al., 2016). This may explain the link between *P. gingivalis*, a heme-dependent pathogen, and T2DM, as well as the association of both with AD (Deshpande et al., 2010). In its attempt to extract heme, *P. gingivalis*, a facultative intracellular microbe, may damage not only cell membranes but also the mitochondrion, triggering a bioenergetic crisis and NLRP3-induced cellular senescence (Bullon et al., 2011). Moreover, age-related brain LPS elevation may trigger intracellular iron migration, an innate immune response to withhold iron from pathogens (Abreu et al., 2018; Ashraf et al., 2018). However, intracellular iron in proximity to redox biomolecules increases the risk of ROS generation, a known trigger of cellular senescence (Lopes et al., 2008; Streit and Xue, 2012). Conversely, the natural iron chelator lactoferrin binds LPS, deactivating NLRP3 (Drago-Serrano et al., 2012; Kruzel et al., 2017; Sfera et al., 2018). Interestingly, lactoferrin was recently identified as an AMP with anti-*P. gingivalis* properties, suggesting a therapeutic benefit in AD (Drago-Serrano et al., 2012; Kruzel et al., 2017).

The aging process, associated with intracellular iron retention, DNA damage, and impaired genomic repair, is a phenomenon we previously defined as ferrosenescence (Sfera et al., 2018). Along these lines, the levels of iron storage protein, ferritin, was found to be a reliable senescence marker, supporting the concept of ferrosenescence (Masaldan et al., 2018). This is in line with a novel hypothesis, suggesting that age-arelated increase in free iron pool resuscitates dormant microbes in the brain parenchyma (Pretorius et al., 2018). Furthermore, intracellular iron can promote Cdk5 nuclear export, tau hyperphosphorylation, and neuronal cell cycle activation (Engmann and Giese, 2009). On the other hand, iron chelation with deferoxamine was shown to have the opposite effect on tau, probably by facilitating Cdk5 nuclear reentry (Guo et al., 2013; Liu J. L. et al., 2018).

## Senotherapeutics: Targeting Senescence in Alzheimer's Disease

Senotherapeutics are pharmacological compounds, aiming at restoring senescent cells to non-senescent status or to trigger their apoptosis and clearance (Olivieri et al., 2018). These agents can be classified into senolytics that selectively eliminate senescent cells and senomorphics that delay or reverse senescence. Recent preclinical studies have shown that senotherapeutics can influence the course of age-related diseases, including AD (Kim and Kim, 2019). The agents described below include novel compounds and repurposed drugs with potential senotherapeutic properties.

### Repurposed Galantamine, Donepezil, Lithium, and Fluoxetine

Galantamine and donepezil are cholinesterase inhibitors widely used in the treatment of AD. They function by inhibiting acetylcholine (ACh)-degrading enzymes and increasing the bioavailability of this neurotransmitter in brain cholinergic tracts. Both drugs were recently demonstrated to protect intestinal barrier and BBB, displaying potential senotherapeutic properties (Nakao et al., 2008; Zhang T. et al., 2015; Zhang Y. et al., 2017; Wazea et al., 2018). ACh-producing intestinal T cells have been reported to promote commensal microbes' immune tolerance, protecting against inflammation and barrier disruption (Dhawan et al., 2016). A recent study showed that alpha 7 nicotinic ACh receptor (α7nAChR) agonists function by deactivating NLRP3 in monocytes and microglia, promoting tolerance to commensals (Ke et al., 2017). Moreover, vagal nerve stimulation and transcranial direct current stimulation (tDCS) may present with senotherapeutic properties as they enhance cholinergic signaling (Chang et al., 2018).

Lithium, a drug used in the treatment of bipolar disorder, was reported to inhibit both mTOR and GSK3β, protecting the ECs of intestinal barrier and BBB (Motoi et al., 2014; Bosche et al., 2016; Steinbach et al., 2017; Martin et al., 2018). In addition, lithium modulates Cdk5, probably stabilizing neuronal cells in post-mitotic state (Jordà et al., 2005).

Fluoxetine, a selective serotonin reuptake inhibitor (SSRI) utilized in the treatment of major depressive disorder was demonstrated to inhibit NLRP3 and SASP, suggesting senotherapeutic properties (Diniz et al., 2016; Du et al., 2016). Indeed, a novel study reported that SSRIs, as a group, decrease the risk of conversion from mild cognitive impairment (MCI) to AD, likely by lowering cellular senescence (Bartels et al., 2018).

### Mitophagy as a Senotherapeutic Strategy

Recent studies have associated defective mitochondria with cellular senescence as defects of these organelles activate NLRP3 (Liu Q. et al., 2018). On the other hand, elimination of defective mitochondria, mitophagy, delays senescence and lowers inflammation. Preclinical studies linked mitophagy enhancement to improved cognition, while accumulation of defective mitochondria was associated with AD pathology (Cai and Tammineni, 2016; Kerr et al., 2017).

Mitophagy as a therapeutic intervention was studied the most in PD in which defective mitochondria are cleared via phosphatase and tensin homolog (PTEN)-induced kinase 1 (PINK1) and the E3 ubiquitin ligase parkin (PARK2) (Pickrell and Youle, 2015). Disruption of this autophagic pathway is a well-established pathogenetic mechanism in PD that may also play a role in AD (Martín-Maestro et al., 2016).

Another mitophagy system, associated with AD and traumatic brain injury (TBI), involves the inner mitochondrial membrane phospholipid, cardiolipin (Chu, 2018; Chao et al., 2019). Externalization of cardiolipin to the mitochondrial surface was shown to activate neuronal mitophagy in rodents (Chu et al., 2013).

Mitophagy-inducing agents currently available include Mito-CP (3-carboxyl proxyl nitroxide), Mito-Metformin, and MitoTam (mitochondria-targeted tamoxifen) (Boyle et al., 2018; Hubackova et al., 2019). These compounds were demonstrated to activate mitophagy by various mechanisms, including depletion of ATP or adenine nucleotide translocase-2 (ANT2) (Singh et al., 2009; Zhang C. et al., 2015). Interestingly, several antibiotics, including quinolones, aminoglycosides, and β-lactams, were found to damage mitochondria, inducing cellular senescence (Kalghatgi et al., 2013; Stefano et al., 2017). Conversely, tetracycline derivatives, doxycycline, and minocycline were associated with the activation of mitophagy in ECs, suggesting protective effects for biological barriers (Dong et al., 2015; Xing et al., 2017).

### Histone Deacetylase Inhibitors as Senotherapeutics

Histone deacetylases (HDACs) are enzymes involved in the epigenetic regulation of gene expression via histone proteins. HDACs have been involved in the pathogenesis of AD, and some HDAC inhibitors (HDACi) may present with cognition-enhancing properties (Xu et al., 2011). HDAC 1 and 2 inhibitors, including valproic acid (VPA), have been demonstrated to correct defective microglial phagocytosis, facilitating the elimination of molecular waste and dead cells (Datta et al., 2018). VPA, a drug utilized in the treatment of epilepsy and bipolar disorder, was recently shown to possess anti-HSV-1 actions, indicating a potential benefit in AD (Crespillo et al., 2016). In addition, this compound prevents LPS-induced ECs damage, protecting intestinal barrier and BBB (Chuang et al., 2014; Kasotakis et al., 2017).

Aside from VPA, other HDACis currently in clinical use include trichostatin A, sodium butyrate, and suberoylanilide hydroxamic acid (SAHA or vorinostat). SAHA is both an HDAC 6 inhibitor and an iron chelator, suggesting senotherapeutic properties (Hwang et al., 2015). SAHA is currently approved for the treatment of advanced primary cutaneous T cell lymphoma, but it also possesses anti-*P. gingivalis* properties, suggesting a therapeutic role in AD and periodontal disease (Yoshioka et al., 2003; Mann et al., 2007). Moreover, VPA and SAHA were recently found efficacious against *Mycobacterium tuberculosis*, an intracellular pathogen, suggesting efficacy against facultative intracellular microbes, including *P. gingivalis* (Rao et al., 2018).

It was recently reported that sirtuin 6 (SIRT6), a protein presenting with HDAC-like senotherapeutic properties, inhibits NF-κB and EC senescence, suggesting AD therapeutic benefits (Lappas, 2012; Zhao et al., 2016).

### Iron Chelators in Cellular Senescence and Alzheimer's Disease

Iron is a pro-growth nutrient that accumulates in senescent cells, contributing to genomic instability and ROS generation (Killilea et al., 2003). A major component of the aging marker lipofuscin, iron is a driver of cellular senescence via mTOR activation and inhibition of mitophagy (Terman and Brunk, 1998; Höhn et al., 2010; Bayeva et al., 2012). Iron chelators, such as deferoxamine, are mTOR inhibitors demonstrated to lower the markers of senescence (Ohyashiki et al., 2009; Inoue et al., 2018). For example, intranasal administration of deferoxamine was found beneficial in animal models of AD, PD, and stroke (Fine et al., 2017). Moreover, as pathogens and host innate immune cells share the same iron pool, iron chelators deny this biometal to both, lowering microbial survival and ROS formation (Thompson et al., 2012). For this reason, iron chelator nanoparticles have been studied as AD therapeutics (Liu et al., 2010).

Another iron chelator with senotherapeutic properties, α-lipoic acid, is a BBB-crossing mitochondrial molecule with beneficial effects in AD (Baeeri et al., 2019; Camiolo et al., 2019). Preclinical studies linked this compound to mTOR inhibition and protection against brain ischemia (Gao et al., 2018). Other recent studies associated α-lipoic acid with intestinal barrier and BBB protection, indicating antitranslocation properties (Schreibelt et al., 2006; Varasteh et al., 2017).

The natural iron chelator lactoferrin, recently identified as an AMP, was found protective of ECs and biological barriers (Krylov et al., 2007; Wu et al., 2014). Inhibiting mTOR signaling and decreasing the iron pool, lactoferrin may be of potential therapeutic benefit in AD (Jenssen and Hancock, 2009; Zhang et al., 2014; van Splunter et al., 2018).

### Inflammasome Inhibitors and Alzheimer's Disease

NLRP3 inhibitors are novel senotherapeutic agents that delay EC senescence and microbial translocation, suggesting beneficial effects in both AD and chronic inflammation (Yi, 2017; Yin et al., 2017; McAllister et al., 2018; Qi et al., 2018). Here, we focus primarily on NLRP3 inhibitors associated with the restoration of biological barriers.

MCC950, a diarylsulphonylurea inhibitor, lowers pyroptosis by selectively blocking NLRP3 inflammasomes, restoring the integrity of intestinal barrier (Fan et al., 2018; Perera et al., 2018). MCC950 also inhibits IL-1β, restoring BBB integrity (Lang et al., 2018). Interestingly, in PD, Cdk5 was shown to activate NLRP3, suggesting that inflammasome inhibitors may lower the detrimental effects of this kinase on neurons, preventing senescence and cell cycle engagement (Zhang et al., 2016). Indeed, to activate cytosolic NLRP3, Cdk5 must exit the nucleus, an event that triggers the neuronal cell cycle. Moreover, MCC950 has been shown to prevent immunosenescence of innate immune cells by blocking *P. gingivalis*-induced pyroptosis (Fleetwood et al., 2017).

INF 39, an acrylate NLRP3 inhibitor, was shown to decrease bowel inflammation in animal models by downregulating IL-1β, suggesting a therapeutic role against microbial translocation (Cocco et al., 2017; Pellegrini et al., 2018).

Milk fat globule membranes (MFGM) were reported to lower bacterial translocation in animal models by inhibiting NLRP3 and increasing the expression of intestinal tight junctions, proteins opposing microbial translocation (Li Y. et al., 2018).

Short-chain fatty acids (SCFAs) were found trophic for IECs, restoring the integrity of intestinal barrier by functioning as energy sources and NLRP3 deactivators (Feng et al., 2018).

Statins were described as protective of ECs in both intestinal barrier and BBB via NLRP3 inhibition, reviving the debate about the benefit of these drugs in AD (Schreibelt et al., 2006; Krylov et al., 2007; Varasteh et al., 2017).

## Conclusions

Commensal gut microbes live in symbiosis with the human host as long as they reside in the GI tract where they can be kept under control. Cellular senescence alters the integrity of biological barriers, allowing translocation and dissemination of gut microorganisms throughout the body tissues, including the brain. Operating “behind enemy lines,” pathogens can gain control of host immune defenses and metabolism, triggering senescence and neurodegenerative pathology.

Senotherapeutics inhibit cellular senescence program, restoring the integrity of biological barriers. Moreover, the recent association of chronic *P. gingivalis* infection with both cellular senescence and AD emphasizes the importance of promptly treating periodontal disease.

Aging, a major risk factor of AD, is associated with senescent cell accumulation and SASP-induced pathology. In the CNS, senescent brain cells may display aberrant traits, including neuronal cell cycle activation and phagocytosis of viable neurons and synapses by aggressive glial cells. Since the molecular underpinnings of senescence, NF-kB-linked NLRP3 assembly, is modifiable, age-related neurodegenerative disorders could be epigenetically, pharmacologically, and immunometabolically influenced not only from within the CNS but also from the body periphery.

## Author Contributions

All authors listed have made a substantial, direct and intellectual contribution to the work, and approved it for publication.

### Conflict of Interest Statement

The authors declare that the research was conducted in the absence of any commercial or financial relationships that could be construed as a potential conflict of interest.
